# Microbial lipo-chitooligosaccharides as key regulators of plant growth: Recent updates

**DOI:** 10.1016/j.biotno.2026.10.001

**Published:** 2026-10-19

**Authors:** Mohammad Miransari, Shirin Adham, Sakineh Moradkhani, Mahdiar Miransari, Amir Arshia Miransari, Neda Doostikhah, Amir Karimi

**Affiliations:** a Department of Book & Article, AbtinBerkeh Scientific Company, Isfahan, Iran; b Department of Biology, Payame Noor University, Tehran, Iran; c Medical College, Na.C., Islamic Azad University, Najaf Abad, Iran; d Masjid Suleiman Agricultural Jihad, Khuzestan, Iran; e Isfahan, Municipality’s Organization for Urban Aesthetics, Landscape and Green Space, Isfahan, Iran

**Keywords:** Cellular morphogenesis, LCOs, Myc-LCOs, Nod factors, Plant-microbe signaling, Root nodules

## Abstract

Chemical fertilization is a favorite method of providing plants with their essential nutrients by farmers. However, due to being subject to leaching and emission of greenhouse gases, the complementary methods of fertilization such as biological ones (i.e. plant-microbes associations) may be economically and environmentally recommendable. During initiation of intimate associations between plants and soil microbes, a set of organic molecules including phenolic compounds such as flavonoids and lipo-chitooligosaccharides (LCOs) are exchanged between symbionts resulting in establishment and development of symbiosis. Due to competition with indigenous soil microbes, microbial inoculants including rhizobium and arbuscular mycorrhizal fungi may not be as efficient. So, interestingly, researchers have used organic molecules including bacterial LCOs, which can positively affect plant growth and yield production. However, more has to be indicated on related mechanisms including identification and expression of related genes in plant and bacteria. The present review article is about some of the most recent in LCOs production, effectiveness, compatibility and marketing. It also has reviewed and analysed how LCOs: 1) are produced by bacteria and mycorrhizal fungi, 2) are perceived by plant, 3) affect plant growth including the roots, 4) may act in stress conditions, and 5) affect climate change. LCOs and soil physicochemical properties are among the most important factors affecting the efficiency and so LCOs commercial production. Such analyses are useful for the development of microbial strains and species with high and effective LCOs production, which can efficiently enhance the growth and yield of legumes and non-legumes and are beneficial for environment.

## Highlights

• Biological methods (plants and microbes) to provide plants with essential nutrients.
• Production of organic molecules by symbionts during the initiation of symbiosis.
• The use of microbial lipo-chitooligosaccharides (LCOs) affecting plant growth.
• More has to be indicated on mechanisms which determine LCOs affecting plant growth.
• These analyses are useful for the development of efficient LCOs-producing microbes.

## 1 Introduction

Chemical fertilization is a quick and favorite method of providing plants with their essential nutrients by farmers. Chemical fertilizers are reliable and stable across diverse environments, an important advantage over biological products, and they currently feed more than half of the world population. However, due to being subject to leaching and emission of greenhouse gases, alternative and complementary methods of fertilization including the biological ones (i.e. plant-microbes association) may be economically and environmentally recommendable.^1^, ^2^, ^3^

Soil microbes can make the plant tolerate the stressful conditions by: 1) increasing the availability of soil nutrients, 2) interacting with the other soil microbes, 3) controlling pathogens, 4) alleviating different types of stress, and 5) production of different organic molecules.^4^, ^5^, ^6^, ^7^ Soil microbes have a wide range of activities including the production of different organic molecules affecting plant growth, human health and the environment. These organic products including chemical signals, consisting of phytohormones, extracellular enzymes and secondary metabolites, play major roles in the growth and activities of microbes including their association with their host plants and helping the host plant to tolerate the stressful conditions. Additionally, these microbial products are used as synthetic chemicals for the production of biofertilizers, biopesticides and bioherbicides.^8^^,^^9^

Plants are able to develop symbiotic associations with different microbial species including bacteria (i.e. rhizobium) and fungi (i.e. arbuscular mycorrhizal fungi).^10^ The establishment and development of these types of symbiosis would require a set of molecular communications between the host plant and soil microbes. Different types of molecules are used by the host plant and the microbial species for the initiation of the symbiosis including phenolic compounds such as flavonoids and lipo-chitooligosaccharides produced by rhizobium (LCOs) and mycorrhizal fungi (Myc-LCOs).^11^^,^^12^ The flavonoids produced by the host plant result in the expression of the bacterial *nod*-genes, and subsequent production of LCOs by the bacteria.^1^^,^^13^

The practical use of organic molecules produced by the host plant for the enhancement of the symbiosis process between legumes and rhizobium has been indicated by research. For example, the use of genistein produced by soybean roots, has been effective on the enhancement of biological N fixation by *Bradyrhizobium japonicum*. Although microbial inoculants are suitable alternatives to the use of chemical fertilization, their efficiency may not be as high due to, for example, the competition with soil indigenous bacteria. So, this is one of the main reasons for the use of microbial products including LCOs for the improvement of plant growth by enhancing microbial association with the host plant. The LCOs can also positively affect seed germination and plant growth.^14^, ^15^, ^16^

So, with respect to the significance of the bacterial LCOs affecting a wide range of activities in the ecosystem, including their complementary use in fertilizing crop plants, in the present review some of the most recent related details have been reviewed and analysed and some future perspectives have been presented.

### 1.1 Microbial products

As previously mentioned, different products, with a wide range of activities, are produced by soil microbes affecting ecosystem health and production. The interesting effects of microbial products including exopolysaccharides^6^, ^17^, ^18^ and LCOs on human health and environmental conditions have been shown by research. These organic molecules are produced by different types of soil bacteria such as plant growth promoting rhizobacteria (PGPR) including *Cyanobacteria* sp., *Pseudomonas* sp., *Streptococcus* sp., *B*. *japonicum*, rhizobium and arbuscular mycorrhizal fungi.^19^

Researchers have determined the enhancing effects of PGPR on plant growth^9^^,^^20^^,^^21^ and soil characteristics. For example, the production of exopolysaccharides by *Cyanobacteria* may improve the properties of soil by the formation of biocrusts. Additionally, during the process of symbiosis, the production of LCOs by the symbiotic bacteria may result in the deformation of root hairs and subsequent formation of root nodules. Accordingly, the LCOs are called Nod factors and can also act as Myc factors during the process of mycorrhizal symbiosis, resulting in the colonization of the host plant roots and improved uptake of water and nutrients.^13^^,^^22^, ^23^, ^24^

Interestingly, the production of LCOs has become commercialized and it is available in the market. Research and technical attempts are being done to improve the efficiency of LCOs formulations available in the market. The bacterial strain *Cupriavidus necator*, with the interesting ability of using CO_2_ as the sole source of carbon and H_2_ as the sole source of energy, has been used for the commercial production of LCOs and other organic compounds. Related to the traditional microbial inoculants, the LCOs have a broader range of applications such as the use for both legumes and non-legumes and for monocotyledon plants.^7^^,^^25^

### 1.2 LCOs functioning

The LCOs contain a backbone of *N*-acetyl-glucosamine with 4-5 residues, their nonreducing end has a fatty acid chain and the specific substitutions on the reducing and nonreducing ends determine their host specificity.^26^ The differences in bacterial *nod*-genes and the different enzymes produced by the bacteria determine the structure and functioning of LCOs (Fig. 1).^27^

**Fig. 1:**
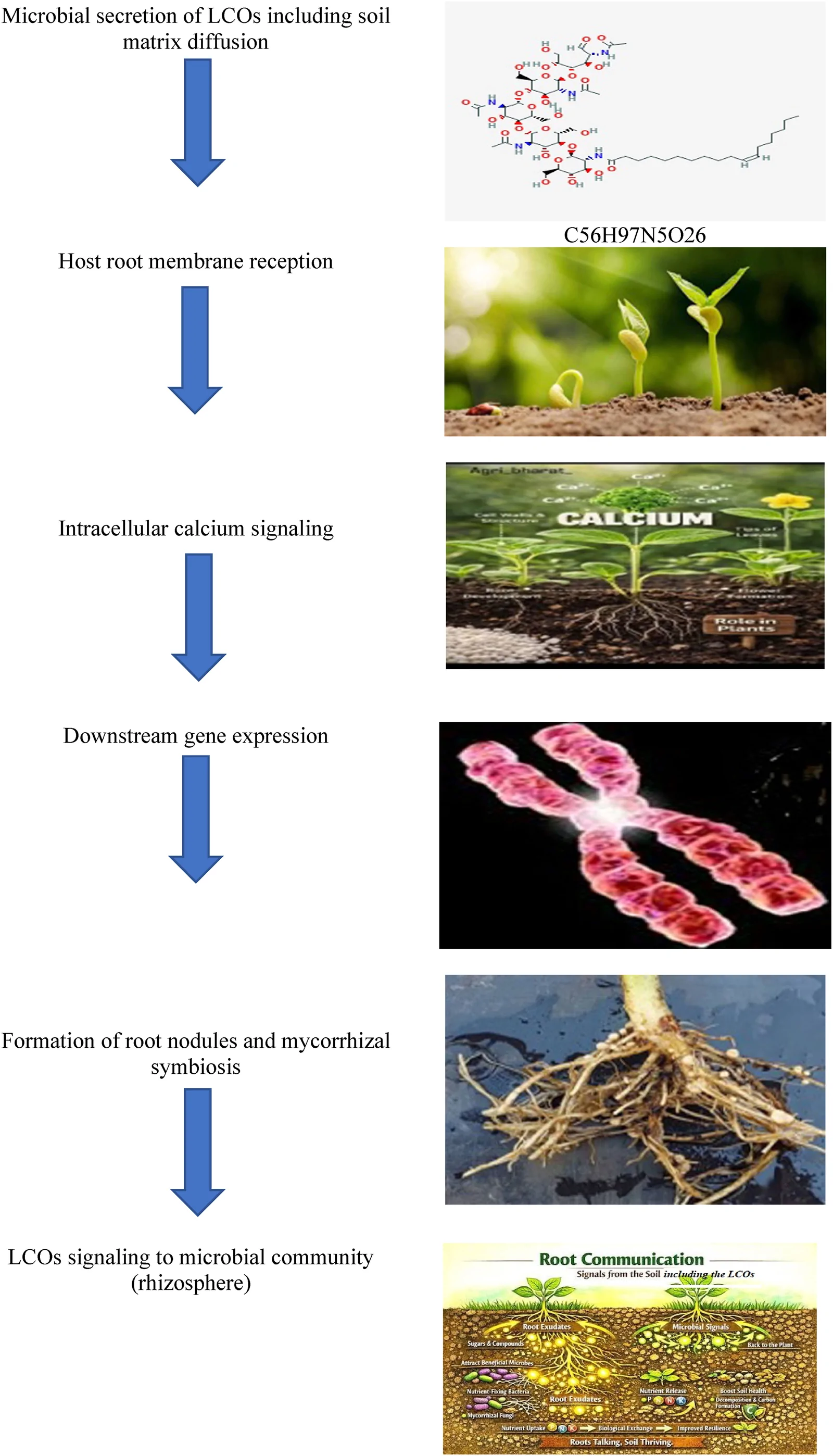
The conceptual framework diagram illustrating the multi-level cascade of the representative LCOs.

There are different parameters, which may affect the ability of the bacterial strain to infect the host plant, among which the interaction between the LCOs and flavonoids and the interaction between LCOs and plant receptors are the most important ones. The LCOs produced by rhizobium are perceived by specific plant receptors resulting in the activation of different molecular pathways and transcription factors. Subsequently, plant genes are activated and plant hormones are produced.^28^, ^29^, ^30^

It has also been indicated that the LCOs not only affect the growth and physiology of plant roots, but also affect the gene expression perhaps in the epidermis. The LCOs are able to activate different plant genes including the ones essential for metabolism, nutrient uptake, cell cycling and functioning, and plant systematic resistance.^31^ Such functioning of LCOs indicates the importance of these molecules for plant health, growth and yield production.^4^^,^^32^, ^33^, ^34^

The LCOs favorably affecting crop yield production in the field has been indicated by research. Although the LCOs are highly bioavailable, their stability and half-life are functions of environmental conditions (climate and soil) as well as plant and microbial species.^35^ These may all affect the biochemical structure of LCO and so their usability in the field. The most optimum conditions for the use of LCOs must be defined for different crop plants in the field so that the highest efficiency of LCOs application is resulted.

### 1.3 Rhizobium and LCOs production

The production of LCOs by rhizobium may be enhanced by two different methods including: 1) the use of flavonoids, which increase the expression of *nod* genes and results in the production of LCOs by rhizobium or 2) by the use of efficient microbial consortium with the ability of excreting higher LCOs from the microbial medium to treat the host plant. The two molecules including genistein and LCOs are commercially produced and used for the enhancement of plant growth, nodulation and biological N fixation.^36^^,^^37^

The LCOs produced by rhizobium may result in the deformation of root hairs and so, formation of root nodules, or may be decomposed by soil microbes or plant host enzymes, or diluted in the soil solution.^33^^,^^38^ The first LCOs, used for the activation of plant genes were produced in the lab by treating rhizobium with genistein resulting in activation of bacterial nod genes and subsequent production of LCOs.^12^^,^^39^ The authors tested the effects of LCOs and gibberellins on the germination of barley seeds and found LCOs as the more effective treatment.

### 1.4 LCOs and mycorrhizal fungi

The symbiosis between mycorrhizal fungi and the host plant is established by the exchange of molecules including chitooligosaccharides (chitin) and LCOs. The lysin-motif-receptor-like kinases (LysM-RLK) is essential for the recognition of chitin and LCOs during the establishment of the symbiosis process and for stimulation of plant immunity. The role of chitin in triggering plant immunity has been indicated by activating the phosphorylation of mitogen-activated protein kinase, production of reactive oxygen species, and expression of defense genes.^23^^,^^40^^,^^41^

However, for the establishment of symbiosis, arbuscular mycorrhizal fungi may inhibit plant immunity by LCOs or production of proteins and effectors, though the mechanisms by which the plant control the activation of symbiosis process and immunity has yet to be indicated.^23^^,^^40^^,^^41^ The role of chitin in mycorrhizal symbiosis with the host plant is more essential than the role of LCOs and as a result the fungi control the activity of LCOs, so that the chitin can result in the development of the symbiosis.^42^^,^^43^

The LCOs are not just produced by mycorrhizal fungi but by other soil microbes including the pathogens. So, the plant must be able to distinguish among different types of LCOs excreted by different species of soil microbes^27^ or the plant would be susceptible to different diseases rather than developing a mutualistic symbiosis with the host plant. This indicates the importance of LCOs for other physiological processes in plants and their associative microbes including: 1) the competence with the other soil microbes for health and resources, and 2) the regulation of plant responses such as the growth of lateral root hairs^44^^,^^45^ for interacting with soil microbes.^46^^,^^47^

For example, rice and barley may have the ability of developing a symbiosis with mycorrhizal fungi in nutrient deficient conditions.^48^ The conclusion by Zhang et al.^23^ was that although plants may recognize identical or similar molecules produced by soil microbes, they may provide different routes so that they can differentiate between immunity and symbiosis, mainly done by the combination of different receptors. Nutrient starvation and not nutrient high levels, regulate the LCOs recognition and perception by plant through NSP1 and NSP2 pathways.^47^

The Nod and Myc factors produced by rhizobium and mycorrhizal fungi, respectively, establish the initial stages of the symbiosis between the two symbionts.^49^ The differences in the biochemical structures of the two factors and their perception by different plant surface cell receptors result in the cascade of different signaling pathways, which proceed with the next stages of symbiosis.^50^

All LCOs contain a backbone of three to five N-acetylglucosamine residues, N-acylated at the non-reducing end. The synthesis of main structure of LCOs in bacteria are determined by the nodABC genes. The N-acetyl-d glucosamine transferase, chitolipooligosaccharide synthase, is produced by the activity of nodC gene combining N-acetylglu cosamine monomers into a single molecule using β-1,4 glycosidic bonds. However, nodB produces deacetylase eliminating acetyl residues at the non-reducing end of the Nod factor. Additionally, the product of nodA gene is N-acyltransferase, attaching a fatty acid at the non-reducing end. These genes are all essential for the process of symbiosis, because, if mutated, the bacteria would not be able to produce Nod factors for the establishment of symbiosis.^51^

Mycorrhizal fungi and rhizobium are significantly different in terms of morphology and the natural traits. However, the early steps in the establishment and development of symbiosis are identical as the two symbionts produce signaling molecules including Nod factors (rhizobium) and Myc factors (mycorrhizal fungi). The backbone of Nod and Myc factors is made of a chitin oligosaccharide backbone consisting of four or five N-acetylglucosamine residues (GlcNAc). The N-acyl group, attached to the non-reducing terminal sugar, results in the production LCOs, while the Myc factors include lipochitooligosaccharides (Myc-LCOs). The two factors are different in terms of structural arrangement as Myc factors do not have an O-acetyl group of Nod factors group at position 6 of the non-reducing end.^52^

During the establishment of rhizobium and mycorrhizal fungal symbioses, plants spent a significant part of their photosynthetic carbon for obtaining N, P and other nutrients from their microbial symbionts.^53^ Although it may be source-consuming for the host plant, it is done on a balanced way so that it would be beneficial for the plant as it obtains its essential nutrients in a more economically and environmentally recommendable manner than chemical fertilization. Once the LCOs are perceived by the surface cell receptors, plant regulates its flux of photosynthetic carbon towards the microbial symbionts so that they would acquire their essential carbon for the establishment and development of symbiosis.^54^

According to Li et al.,^47^ interestingly, the activation of symbiosis signaling in cereals is done by rhizobia LCOs though the specificity of LCO perception in legumes is higher than cereals. Nutrient starvation activates LCO perception in plants by the nodulation signaling pathway, which is transcriptionally regulated resulting in the expression of LCO receptors. So, nutrient starvation is a way by which the production and perception of LCO is done resulting in the enhanced engagement of rhizobium and mycorrhizal fungi, which is of significance in legumes and cereals. The above-mentioned mechanisms indicate how the plant may regulate its association with the microbial symbionts during the LCO perception so that optimal symbiotic interactions are resulted, with benefits for the microbes and the host plant.

### 1.5 LCOs perception by plant

The presence of receptors on the surface of plant roots determines the way by which plants respond to the organic molecules produced by soil microbes regulating plant functionality including immunity and symbiosis.^55^^,^^56^ Among plant receptors, pattern recognition receptors can differentiate between beneficial and pathogenic soil microbes and so enhance plant immunity responses.^57^ However, the activation of these receptors may decrease plant growth and as a result it is of significance to investigate how the activities of such receptors may be regulated by plant.^58^^,^^59^ So, the activation of the receptors during the establishment of plant-microbes symbiosis must be regulated by the host plant resulting in the minimum burden for plant.

The important role of Nod factor receptors is to allow nodulation in the presence of rhizobium. The LCOs are recognized by plant receptors, which determine if the LCOs are produced by the pathogenic or the symbiotic bacteria.^57^ During the symbiosis process a blend of LCOs are produced by the bacteria, which are perceived with highly specific receptors.^50^^,^^60^ So, highly developed reception systems can make the plant respond properly to the exertion of organic molecules by soil microbes. The unique harmony among plant receptors, functional genes, and signaling pathways is essential so that the plant can react promptly in the presence of soil microbes producing LCOs.

Fabaceae plants including *Medicago truncatula* and *Lotus japonicus* perceive LCOs with LysM-RK receptors, as the essential components for the symbiosis with rhizobium, with the ability of recognizing LCOs with high affinity.^27^ Additionally, arbuscular and ectomycorrhiza also produce LCOs for the process of symbiosis with their specific host plant.^61^ The LysM-RK chitin receptors determine plant immunity potential and symbiosis, and the small and large chitins are also able to act in the symbiosis process.^42^ However, in the case of fungi, a wide range of such microbes including the symbiotic, pathogenic and saprophytes are able to produce LCOs.^46^

The recognition of the LCOs acting as symbiotic or immunity molecules is done by the LysM-RKs receptors, though its related details have yet to be indicated. However, the physiological conditions of the host plant may determine if the LCOs molecules may act as symbiosis or immunity molecules.^47^ Interestingly, in phosphorus deficient conditions the act of LCOs as immunity molecules may diminish, making the LCOs act as symbiotic molecules with the microbiota in plant rhizosphere.^47^^,^^62^

So, plant recognition of LCOs is one important step in the establishment and development of plant-microbe-associations. Understanding the mechanisms by which plant recognize and utilize LCOs may help researchers modify the structure of LCOs. Accordingly, LCOs would be perceived by the host plant more efficiently and this would make the commercial production of LCOs, more likely.

### 1.6 Can LCOs act as broad-spectrum cross-kingdom signals?

Plant receptors determine how microbial products are perceived by plant cellular surface resulting in the production of different signaling cascades. There is a high diversity in the spectrum of ligand recognition by the receptors of plant cell surface.^63^ Understanding such a diversity may be achieved by investigating the natural variation in the receptors of plant cell surface.^64^ The specific indication of ligand-binding determined by molecular and structural characteristics, and the way by which plant receptors control different signaling pathways, are of significance. Further details in this respect may result in the breeding crop plants with higher immunity and symbiosis potential.

For example, CERK1 (chitin elicitor receptor kinase 1), which is a LysM-RLK and was initially found in Arabidopsis affecting plant defense against fungi, is one of the main plant receptors, controlling plant-microbe’s interactions. Microbial LCOs are determined by LysM-RLKs Nod factors resulting in the signaling cascade of symbiosis in *M*. *truncatula*. In addition to legumes, the central acting of LCOs in a few non-legume plants including Arabidopsis, rice, corn and cotton^65^ has been detected. Interestingly, CERK1 can also benefit this type of plant-microbe interactions by the colonization of arbuscular mycorrhizal in *M*. *truncatula* and rice. For example, the simultaneous perception of LCOs and CERK1 in *M*. *truncatula* results in the process of symbiosis.^66^

However, it has yet to be indicated, though CERK1 is versatile, how it may act specifically in plant-microbe interactions.^49^ Different parameters determine symbiosis specificity such as the composition of receptor complex, gene expression and the structure and length of ligand.^27^ For example, the immunity of rice is by OsCEBiP-CERK1 signaling, while its symbiosis is by OsMYR1-CERK1 signaling. The receptor itself also determines its specificity, for example, the CERK1 homolog CERK6, and Nod factor receptor result in LCO-binding.^66^ Further research is essential to indicate how LCOs may also be perceived by non-legumes and results in the signaling cascade, which for example, affect plant immunity and interactions with soil microbes.

The LCOs may indirectly affect soil microbiome by affecting the physiological properties of the host plant. Depending on the type of the associative microbes, the LCOs may induce symbiotic or immunity responses in the host plant. Accordingly, such responses result in different types of interaction between plant roots and the microbiome in rhizosphere resulted by root exudates.^67^ This is so another important aspect, which deserves more research to determine how LCOs may indirectly affect the microbiome of rhizosphere. The topic is of significance, because the alteration of rhizosphere by root exudates significantly affects the ecosystem efficiency. The other important research topic is the direct effects, which LCOs may have on non-target microorganisms as the evidence on the topic is constrained. The topic is of significance for the production and development of microbial consortium, which produce signal molecules, used for the inoculation of legumes and non-legumes.

### 1.7 Production of efficient microbial LCOs

The most important topic about LCOs is their efficient use in the field by the growers, while economically and environmentally recommendable. LCOs structural and physiochemical properties affect plant responses to the LCOs and subsequent plant growth, symbiosis and immunity.^67^ Additionally soil physicochemical properties not only influence the diversity of soil microbes, but also the utilization of LCOs by plant.^68^

Research must find the most efficient microbial strains, which are capable of producing stable structure of LCOs in the soil to make the commercial production of LCOs more likely. The LCOs are subjected to volatilization, microbial degradation and other reactions affecting their stability and subsequent utilization by the host plant.^69^ So, researchers may look for the optimum conditions for the production and storage of LCOs, while economically affordable. Interestingly, plant receptors shape the community of soil microbes,^67^ which may be a cue for breeding plant species, which can attract the microbial species with the ability of producing the most efficient and stable LCOs.

### 1.8 LCOs and plant growth

Although the important role of LCOs in the formation of nodules have been indicated, these molecules may have other activities in legumes and non-legumes including alteration of morphogenesis, gene stimulation, and cell cycling (mitosis division).^34^ The Nod factors result in membrane depolarization of root hairs by distributing Ca^2+^ in the cytoplasm of root hair tip. Such mechanisms may indicate how the LCOs are able to enhance seed germination.^12^^,^^39^^,^^70^^,^^71^

The LCOs enhances the formation of root hairs, nodulation, nitrogen and chlorophyll content, and photosynthesis rate, collectively resulting in the enhanced growth and yield production. The other experiments have also indicated how the LCOs may increase the activity of nitrogenase and the efficiency of photosynthesis, and nodule number.^72^, ^73^, ^74^

The positive effects of LCOs have also been indicated in field conditions.^75^ Although in severe drought or waterlogging conditions the effects of LCOs were not apparent, in the moderate level of drought stress the positive effects of LCOs on crop yield were observed in the field. It has been a while since the commercialization of LCOs has been done for treating seeds or spraying the leaves, which can increase root inoculation with bacteria and plant uptake of N, P and K.^25^^,^^76^

The use of LCOs has been effective on the alleviation of water stress in soybean fields.^77^^,^^78^ The *Arabidopsis* plants treated with LCOs had high number of morphological alterations including the enhanced number of root tips and root and leaf area.^65^ The LCOs treatment resulted in the higher distribution of Ca^2+^ and inhibited the production of reactive oxygen species in corn, *Arabidopsis*, soybean and tomato.^44^^,^^79^ The enlargement of plant roots enhances plant ability for higher water and nutrient uptake; however, this enlargement is up to a level as the plant is able to self-regulate the growth of the ground- and above-ground parts.^80^ So, plant may recognize and utilize microbial LCOs up to the level, which does not harm plant growth and yield production.^37^

The *Arabidopsis* and soybean plants treated with LCOs in salt stress conditions had a higher production of antioxidant enzymes.^63^^,^^81^ The LCOs treatment resulted in expression of stress genes and the genes essential for the enhanced growth of root growth.^5^ The LCOs also significantly enhanced the production of plant hormones in plants, along with the enhanced formation of lateral roots. Similarly, a higher seed germination and root growth was observed in the seedlings of cotton, barley, and rice.^12^^,^^13^^,^^22^ Corn growth and photosynthesis in control and salt stress,^72^ tomato plant flowering and yield,^65^ canola root growth^82^, ^83^ and buckwheat flavonoid production was increased by the use of LCOs.^84^

Marburger et al.^85^ examined the effects of LCOs on the development of sudden death syndrome (SDR) and Scleractinia stem rot (SSR) in soybean. Their results did not approve their first hypothesis since LCOs did not affect SDR in soybean, however, their results approved their second hypothesis, because the application of LCOs intensified SSR in soybean. Earlier research has indicated the controlling effects of rhizobium producing LCOs on the development of SDR, however, the single use of LCO, which is not commercially available, would not cause such effects. The rhizobium negatively affects the growth of *Phytophthora* sp. and so, may control the disease.^86^ Such results indicate the proper concentration of LCOs, which may improve plant growth and yield production, while controlling different diseases for different crop plants, have yet to be indicated by research.

### 1.9 LCOs and root growth

Researchers have indicated the effects of LCOs on root growth. Herrbach et al.^34^ indicated the synergistic interaction of LCOs and auxin on the formation of lateral roots in *Medicago truncatula* by affecting the expression of symbiotic genes. The authors accordingly indicated that the perception of LCOs by *M*. *truncatula* stimulate auxin responses in plant affecting the symbiosis process. It has been indicated that the LCOs may modify the production of auxin in root hairs affecting the infection process by rhizobium.^87^, ^88^, ^89^

Buendia et al.^13^ also investigated the molecular mechanisms, which may stimulate the formation of lateral roots in *Brachypodium distachyon* by LCOs and auxin. For this purpose, the authors determined the formation of the lateral roots in the model plant (monocot) *B*. *distachyon* affected by LCOs and auxin by referring to phenotyping properties, auxin imaging and quantification, and the expression of the auxin homeostasis genes.

Buendia et al.^13^ indicated that LCOs and little concentration of IBA (indole-3-butyric acid), as the precursor for auxin, resulted in the stimulation of lateral root formation by increasing the production of plant auxin content and regulating the expression of auxin homeostasis genes. The conclusion was that the interaction of LCOs and auxin can affect root architecture by regulating auxin homeostasis.^13^ The stimulating effects of LCOs on the root growth of rice, maize and *Setaria* have been shown by research.^5^^,^^48^ The plant hormone, auxin, produced in the aerial parts of the plant, controls root growth and development including the formation of lateral roots in monocots and dicots.^90^

### 1.10 LCOs and stress

The LCOs act as bacteria to plant molecules enhancing plant growth and activities by upregulating the expression of different genes in different conditions including stress. Interestingly, the LCOs in stress conditions may downregulate the activities of some genes and result in the allocation of resources for plant growth instead of plant responses to the stress.^80^ The LCOs are able to affect plant biomass, root growth and branching, embryogenesis, cellular activities and growth, and seed germination.^4^^,^^5^^,^^91^

In stress conditions, plants may use different physiological mechanisms for the alleviation of the stress by scavenging reactive oxygen species.^92^ The use of biological elicitors such as LCOs may enhance plant ability to tolerate stressful conditions. The LCOs are able to increase the activities of antioxidant enzymes and non-enzymatic antioxidants, which are able to scavenge reactive oxygen species in stress conditions. Accordingly, the LCOs in such conditions decrease oxidative stress, by increasing plant antioxidant and photosynthetic activities, and affecting the level of malondialdehyde.^33^^,^^67^^,^^93^

Flavonoids and phenols have antioxidant activities in plants under stress. However, in LCOs treated tomatoes, the response of plant was different as other molecules including glutathione, ascorbic acid and tocopherols alleviated stress conditions on plant growth. Additionally, the LCOs were able to decrease the level of malondialdehyde and increase photosynthetic activities.^93^ In stress conditions, the addition of flavonoids including genistein can alleviate the stress by the initiation of the symbiosis process including the production of LCOs by the associative rhizobium.^94^

### 1.11 Interactions of LCOs with other signaling molecules

The interactions between soil microbes including rhizobia and mycorrhizal fungi, during establishment of symbiosis, may affect symbiotic efficiency. Accordingly, one important aspect, which may significantly affect the functioning of LCOs during their field application is LCOs interactions with other signaling molecules including flavonoids (i.e. genestein) co-applied or produced by the indigenous soil bacteria.^2^

Due to the structural similarities between Nod and Myc factors, they are able to activate the essential signaling of common symbiosis pathways^60^ resulting in root nodulation and mycorrhizal root colonization and subsequent morphological alterations (i.e. root branching). This may be a suitable practice for the sustainable production of crop plants. The enhancing effects of LCOs resulting in a higher growth and yield production in legumes and non-legumes indicate the broader applicability of LCOs than the traditional rhizobia inoculants.^25^

This has paved the way for the appearance of newer biostimulants containing both Nod and Myc factors in the market for the more efficient production of crop plants. Accordingly, this shows the potential advantages of new biostimulants containing the combination of different signal molecules, which may help overcome some limitations such as the presence of indigenous soil rhizobium. The new biostimulants may have the following advantages: 1) they increase the number of nodules and also improve plant morphological and physiological properties, 2) they can be used singly or in combination with traditional rhizobium inoculants, 3) they are applicable for the use with non-legumes, and 4) they can be applied foliarly or as seed dressing.^25^ Future research may look for the interactions between the other signal molecules including exopolysaccharides and volatile organic compounds for the development of multi-functional synthetic communities (SynComs) and smart bio-fertilizers.

### 1.12 LCOs and climate change

Due to the process of global warming resulting in climate change, the use of new technology is essential so that the effects of climate change, unfavorably affecting crop production, are diminished. Additionally, the use of proper agricultural practices can significantly alleviate the unfavorable effects of climate change.^95^ The one important process, which may be affected by plant microbial association is climate change. Agriculture can also significantly affect the process of climate change due to the production of greenhouse gases, which is resulted by the use of chemical fertilization (produced by fossil fuels) and herbicides,^96^ deforestation, and animals and manure. The use of symbiotic microbes including rhizobium and arbuscular mycorrhiza is one of the effective methods,^91^ which may alleviate the negative effects of climate change by the production of secondary metabolites including LCOs.^97^^,^^98^

It is because the production of natural fertilization such as N for the use of plants can significantly decrease the use of chemical fertilization and so the production of greenhouse gases. Accordingly, since the microbial products including LCOs and flavonoids play important roles in enhancing plant growth and the process of microbial symbiosis, they can also alleviate climate change unfavorably affecting crop production in different conditions including stress. So, this is another important reason, which necessitates the commercial production and use of secondary metabolites including LCOs products for alleviating the negative effects of climate change.^99^^,^^100^

## 2 Conclusion

The investigation of microbial products affecting the ecosystem functioning is of utmost significance. The present review highlights some of the most recent in LCOs production, functioning, marketing, and compatibility affecting different ecosystem processes including plant physiology, growth and yield in control and stress conditions. Some of the most updates about the role of LCOs in plant growth including the role of LCOs in the alleviation of stresses have been presented. The interaction of LCOs with other signaling molecule such as flavonoids may also affect their functioning in plant-microbial associations. The role of plant cell surface receptors in the perception of LCOs may distinguish among soil beneficial and pathogenic microbes. Additionally, the role of LCOs in the growth of non-legume plants has also been indicated. The present details indicate the significance of LCOs for the ecosystem emphasizing the need for the commercial production of LCOs to be used more frequently by the farmers. Although microbial inoculants of plant growth promoting rhizobacteria (PGPR), rhizobium and mycorrhizal fungi are frequently used by the farmers, due to some issues such as the competition with the soil indigenous microbes, they may be less effective. So, researchers have found the organic molecules, being exchanged during the association between plants and soil microbes, can more efficiently affect plant growth and ecosystem functioning. Some of the most related recent in this respect has been highlighted.

## CRediT authorship contribution statement

**Mohammad Miransari:** Writing – review & editing, Writing – original draft, Supervision, Methodology, Investigation, Formal analysis. **Shirin Adham:** Conceptualization. **Sakineh Moradkhani:** Investigation. **Mahdiar Miransari:** Conceptualization. **Amir Arshia Miransari:** Conceptualization. **Neda Doostikhah:** Investigation. **Amir Karimi:** Investigation.

## Data availability

All available data have been presented in the manuscript.

## Funding

There was not any funding for the present research.

## Declaration of competing interest

The authors declare they do not have any conflict of interest.
